# New Views of the Process of Defecation, and Their Application to Diseases of the Stomach, &c.

**Published:** 1833-07-01

**Authors:** 


### Medico-Chirurgical Review,

### No. XXXVII.

APRIL 1 to JULY 1, 1833.

### I.

New Views of the Process of Defecation, and their Application to the Pathology and Treatment of Diseases of the Stomach, Bowels, and other Organs; with an Analytical Correction of Sir Charles Bell's Views, &c. By *James O'Beirne*, M.D. Surgeon Extraordinary to the King, and one of the Surgeons of the Richmond Surgical Hospital in Dublin, &c. Octavo, pp. 286. Dublin and London, February, 1833.

Originality, whether theoretical or practical, has been so rare, of late years, in medicine, that it is quite refreshing to see even the pretension to it confidently made. The recent epidemic, indeed, gave origin to some novel doctrines and practices, but unfortunately they were very ephemeral, giving way to one another, like the waves of the ocean. The venous injection promised, for a moment, to realize all the miracles of Medea's cauldron, but soon experienced the common lot of man and his works. The author of the work before us appears to apprehend much opposition from "that tendency of the mind to be slow in admitting, and active in resisting every new truth, particularly whenever that truth happens to conflict with either long-established or preconceived opinions; and shews the sources from whence I chiefly anticipate opposition." We put it to Dr. O'Beirne's cool judgment whether this opposition is not rather against new *doctrines* than against new *truths*, and until every new assertion is proved to be a true one, we think the caution extremely salutary. What has occasioned all this backwardness to receive new truths? Nothing originally inherent in human nature—nothing but the woeful experience that nineteen new truths out of twenty are no truths at all! How can we know that an author is the exception to the general rule till his truth is tried by experience; and who can wonder at the backwardness of the public to make the experiments, when that public is certain of being misled nineteen times in twenty. We cannot but think that Dr. O'Beirne's apprehensions are groundless; for in these days no proposition, however absurd, will long remain untested.

As for opposition, it is the best thing that can happen to any novel proposal. Did opposition prevent the introduction of cool air in small-pox—or the practice of vaccination—or, in fact, any real improvement in medicine or surgery? Certainly not. It is doubtful whether the circulation of the blood would now be known, had not the violent opposition to Harvey secured the discovery against neglect and oblivion. If Dr. O'Beirne's new views be correct, and his practices successful, he ought to pray every night, on his bended knees, for every species of opposition—nay, for every term of reproach from that race of critics which he now objurgates with quotations from Locke. But we shall now proceed to do the author the best service in our power—that of making the contents of his work known to the public.

In the Autumn of 1821, the author treated a case of traumatic tetanus with success by means of tobacco enemata. In May, 1822, he employed the same remedy in another case, but it failed. In both cases, however, he observed that there was great difficulty in pushing the injection higher up than the rectum. This circumstance convinced him that the muscular coats of that gut participated in the state of general spasm. The idea therefore occurred of introducing an elastic tube into the sigmoid flexure of the colon. In October of the same year, an opportunity occurred of testing this idea. The case was one of traumatic tetanus. A large gum elastic catheter was pushed up with great difficulty through the contracted parietes of the intestine; and at length, when introduced to the extent of nine or ten inches, it rapidly passed forward, as through a narrow ring, "when an escape of flatus and fluid fæces took place from its extremity, (the inner end having been cut off and smoothed,) giving great relief to the patient." It was now in Dr. O'B.'s power to administer the tobacco enema, so as to insure its own peculiar effects, and open the bowels. The patient recovered; "and from this period may be dated my unexampled success in treating this hitherto most fatal and intractable disease." In the mean time, other diseases, attended with constipation, came before him, and suggested the trial of a similar remedy—or rather a similar mode of throwing up various purgative fluids. "In almost every instance in which these trials were made, the plan was attended with the most decided and prompt success." Dr. O'B. was also led to the conclusion that, "the natural action and state of the bowel (rectum) were directly opposite to what they have always been considered to be." Experiments were made on the rectum of persons in health, and the author's were confirmed.

"From the earliest period to the present, all physiologists have described the fecal matter as passing freely from the sigmoid flexure of the colon into the rectum, and gradually distending the latter until, by its pressure, such a sense of uneasiness is communicated to the sphincter ani and muscles of the perineum, as to rouse the diaphragm and abdominal muscles to effect its expulsion from the body. It is a universally received opinion also, that the power of retaining the feces and controlling their discharge, depends exclusively upon the sphincter muscles of the anus.

These opinions, it is obvious, originated from the circumstance of the sigmoid flexure and the rectum appearing in the dead body as one continuous tube, and also from the fact of there being nothing like a sphincteric arrangement of fibres observable in the muscular coat of either of these portions of the intestinal canal. But venerable as they are rendered by time, and plausible as they may appear, the following facts and observations will be sufficient to shew that they are quite erroneous, and formed upon the most superficial and deceptive views." 3.

The design of Nature (observes our author) to convert the large intestines into a depôt for the reception and retardation of the fæces would have been rendered abortive, if a free communication existed between the sigmoid flexure and the rectum—such a free communication necessarily exposing the rectum to frequent accumulations, and the sphincter ani to continued irritation.

" *Secondly*. The circumstance of Nature forming one of her chief depôts for excremental matter in a part of the intestinal canal so close to, and continuous with the rectum, as the sigmoid flexure is, appears altogether inconsistent with the idea of a free passage between these portions of the canal.

*Thirdly*. In the act of receiving an enema, every person is sensible of a *considerable degree of opposition to the ascent of the fluid in the rectum*. It is well known, also, to those in the habit of administering injections per anum, that, although the syringe may be in the best order, properly filled, and its pipe fairly inserted up the rectum, *considerable force* is generally required to discharge the fluid, from the resistance given to its passage upwards.—These facts would lead us to infer that the rectum, so far from being open, is firmly contracted and closed.

*Fourthly*. Surgeons find it necessary to pass a finger up the rectum, either to direct the course of a catheter, sound, or staff, to discover whether a fistula communicates or not with the bowel; to detect the presence of a calculus in the bladder, or a stricture in the intestine itself; to ascertain the state of the prostate gland; and for various other purposes; and yet it is a fact that it has exceedingly rarely happened, that, on any of these occasions, the finger has encountered either solid or fluid feces in the rectum, or presented a soiled appearance when withdrawn. Indeed, as far as my experience and inquiry has enabled me to speak on the point, in the few instances in which such examinations have detected the presence of excrement in the healthy rectum, it has been invariably found in very small quantity, and never in any but the lowest part, or pouch, of this intestine. It is, also, a fact familiar to apothecaries and nurses, that the pipe of the injecting syringe, however long it may be, is rarely, if ever, found soiled with fecal matter when withdrawn after administering an enema. These circumstances shew that the rectum is contracted and closed, so as to prevent free communication between it and the sigmoid flexure." 5.

Before proceeding further, we must beg to enter our protest against the correctness of the *third* proposition in the above extract. We appeal to universal experience, whether this difficulty in throwing up enemata be not the *exception*, rather than the general rule. Excepting in morbid irritability of the bowel, or accumulations in the rectum or sigmoid flexure, we rarely experience any difficulty in throwing up fluids, after the pipe has fairly passed the sphincter ani. We confess, therefore, that this early attempt to strain an exception into a general rule in order to support a theory, has thrown us a little on our guard, in this early stage of the examination on which we are entering. At the same time, we do not deny that the *natural state* of the rectum, as of all hollow muscular tubes or pouches, is contraction, *till* distended by their natural contents. Is not the bladder in a state of contraction, till distended by the urine from the kidney? But when distended and containing urine, is the bladder not also in a natural state—that is, in a state which Nature designed it to be in, much more frequently, and for a much longer space of time, than in the condition of contraction? Because the stomach is in a state of contraction (comparatively) in hunger, is it in an *unnatural* state during the digestion of our food, when distended with aliment?

The *fourth* proposition is ingenious. Every practitioner well knows that, generally speaking, when operations are performed on the bladder or rectum, the bowels are previously cleared by an aperient or a lavement; and so far the author is correct, when he says that the finger rarely encounters solid or fluid fæces in the rectum. But, if this preliminary caution is not used, we venture to assert that the finger will *very frequently* come out of the rectum soiled with fæcal matters. In fact, we mean to assert that, except after evacuations, natural or artificial, it is almost as common to find fæces in the rectum as water in the bladder. At the same time, we believe and know that the rectum is not the sole depositary of fæcal matters; for we are quite certain, from much attention to the subject, that *during* almost every natural or artificial evacuation of the bowels, a considerable portion of the evacuated matters comes from the sigmoid flexure of the colon. Any person, who is not very corpulent, may ascertain the fact, by pressing the fingers of his left hand deeply into the hollow of the left ilium, just before an evacuation. He will there feel a kind of wedge, which disappears during the evacuation of the fæces. Nay, we can assure our readers that this very pressure of the fingers will often, we had almost said *generally*, excite the bowel into action, and greatly assist the expulsion of the fæces, in costive habits.

"*Fifthly*. Membranous filaments have seldom, if ever, been found traversing in various directions the cavity of either the small intestines, the cæcum, or the colon, while they have often been met with in the rectum. This fact proves that the parietes of the rectum must have been contracted, and its lining membrane in close contact at all points, for a time sufficient to effect the firm organization of these filaments, and, consequently, that there could have been no communication between this intestine and the sigmoid flexure for, at least, several hours.

*Sixthly*. The two sphincter muscles of the anus are considerably weakened in the disease called prolapsus ani;—in the operation for fistula in ano, these muscles are completely divided, and thereby wholly incapacitated, for a certain time, from acting as sphincters;—not only these muscles, but also a portion of the rectum above them, are occasionally destroyed by venereal, cancerous, and other ulcerative processes; yet it rarely happens that the power of retaining the alvine contents is found to be at all impaired in any one of these cases. It is therefore manifest that this could not possibly occur if the passage into the rectum were as free as it is supposed to be, or if the power of retaining the fæces, and regulating their discharge, depended solely on the sphincter muscles of the anus." 6.

The fifth proposition we do not clearly comprehend, and, therefore, we shall not comment upon it. If Dr. O'Beirne had ever had the misfortune to be cut for a fistula, he would have experienced the disagreeable inability of retaining the fæces *till* the muscle had recovered its power by the healing of the cut. Has Dr. O'B. never seen one of those melancholy cases where the sphincter ani is lacerated in child-birth, and where the inability to retain fæces continues for life? If he has not, we can shew him one whenever he pleases to see it. The reason why, in such cases, the fæces are not *constantly* draining from the rectum appears to be, that they are not *constantly*, but periodically moving along the line of the colon. Once or twice in the 24 hours, the fæces become accumulated in the colon (chiefly the sigmoid flexure) and the rectum, and then the stimulus of their presence induces both portions of gut to contract and expel them. That they very often stagnate, as it were, in the rectum, is well known to those who are often in the habit of examining the vagina of the female. Nothing is more common than to find the rectum loaded with solid fæces in such cases. We shall now give the author's description as to the natural and ordinary state of the rectum.

" *Seventhly.* Seeing the forcible nature of the foregoing facts, and anxious to test the truth of the inferences drawn from them, I have been led to examine the rectum of a number of healthy persons, healthy at least as far as the bowels were concerned, at different times in the same day, in order to ascertain its actual state, and as nearly as possible the time and manner in which it is filled. I proceeded in the following manner, and almost invariably obtained the following results. On passing a stomach tube to the height of half an inch up the rectum, neither flatus nor feces escaped through it; passing it up about an inch and a half higher, it was still found that nothing escaped, but that it could be moved about freely in a space which, on introducing the finger, was ascertained to be what anatomists call the pouch of the rectum, in a perfectly open and empty state. From the highest part of the pouch to the upper extremity of the bowel, generally a distance of from six or seven to eight inches, it was found that the tube could not be passed upwards without meeting with considerable resistance, and using a degree of force sufficient to mechanically dilate the intestine, which was plainly felt to be contracted so as to leave no cavity for this extent. When the instrument reached, in this way, the uppermost point of the rectum, the resistance to its passage upwards was felt to be sensibly increased, until at length, by using a proportionate degree of pressure, it passed forward rapidly, and as if through a ring, more or less tight, into a space in which its extremity could be moved with great freedom; and as instantly a rush of flatus, of fluid feces, or of both, took place through the tube. In some instances, indeed, it happened that neither gaseous nor liquid matter escaped at this moment, but, in all these, the distinct feel of the extremity of the tube having entered a solid mass in the flexure, was communicated to the hand; the instrument, on being withdrawn, exhibited a few inches of its upper extremity covered, and its eyes plugged up, with solid excrement; the person generally went to stool soon after, and passed a large quantity of solid feces. In every instance where the tube presented the least appearance of feces after being removed, this appearance was confined to that portion of its upper extremity which had entered the sigmoid flexure.

In this way, I have also examined the rectum of healthy persons in a few minutes after they had passed a stool, and of others at the moment when they felt a moderate inclination to go to stool, and have ascertained that the rectum is in a perfectly empty and contracted state at both of these periods.

The results of these examinations establish the correctness of the inferences drawn from a number of facts, but in a much more positive and precise manner, for they distinctly prove, first, that in the healthy and natural state, all that part of the rectum above its pouch, is at all times, with the single exception of a few minutes previous to evacuation of the bowels, firmly contracted and perfectly empty, at the same time that the pouch itself and also the sigmoid flexure of the colon, are always more or less open and pervious; lastly, that the sphincter ani muscles are merely subsidiary agents in retaining the feces." 8.

Should these statements be confirmed by the experience of others, they will strengthen very much the views of the author. Dr. O'B. next proceeds to shew, from anatomy, that the parts in question cannot be in any other than the condition above-mentioned. The anatomical reasons we must condense as much as possible. *First.* The small intestines have hardly more than one muscular coat; and the same of the colon and cæcum, with the addition of three narrow longitudinal bands. The rectum possesses an internal coat composed of strong fibres circularly arranged, and also an external one of longitudinal arrangement, the three longitudinal bands of the sigmoid flexure sending down strong fleshy fibres to be expanded upon, and intermixed with those of the external coat. Hence the author concludes the rectum exceeds every other part of the intestinal canal in muscular power. *Secondly,* This is the only portion of gut into which we can trace branches from the regular spinal nerves without previous interlacements with filaments from the sympathetic—consequently it is the only part of the intestinal canal receiving, directly, both motific and sensific nerves. It is fair to infer that this gut then possesses a higher degree of irritability and sensibility. When a tube so constituted is called into strong action, the effect will, he thinks, be perfectly similar to what takes place in the œsophagus after deglutition, namely, to contract its parietes and obliterate its cavity in that portion which is above the pouch. That the rectum is quiescent during the intervals of defecation, is, he thinks, incontrovertible.

"But I shall now show that a change which the flexure undergoes at the same moment, maintains the rectum in this favourable situation until such time as another evacuation of the bowels is about to take place. This change consists in the inferior and greater portion of the empty flexure falling into the pelvis, hanging doubled over, and rather to the left of the rectum, remaining in this situation until it is raised by distension into the place it had previously occupied in the left iliac fossa; and it is scarcely necessary to observe, that the first of these changes of position is one which would effectually prevent the descent of fluid or solid feces, if not of flatus, and thus secure the undisturbed condition of the rectum." 13.

This last statement needs confirmation by others. The author is now prepared to exhibit his own view of the process of defecation, and we shall give them in his words, since claimants to originality are generally jealous of any misconception of their discoveries.

"The contents of the stomach having passed through the pylorus, and entered the superior transverse portion of the duodenum, this portion of the intestine, previously in a passive state, is now roused into activity by the stimulus of distension, the circular and a few comparatively very minute longitudinal fibres which compose its muscular coat, contract forcibly upon the contained mass, and urge it into the next, the middle or perpendicular portion, in which its presence also excites contraction; and thus by a succession of similar dilatations and contractions, the mass is propelled in a gradual and regular manner through the inferior transverse portion into the jejunum, and thence to the termination of the ileum. This process, however, is considerably assisted by the firm, equable, and constant pressure to which the small intestines, in particular, are subjected by the diaphragm behind and the abdominal muscles before in their alternate contractions to assist in carrying on ordinary respiration. It is also greatly facilitated by the circumstance of the gaseous matter necessarily taking the precedence, dilating the bowel before it, and thus, if not wholly effacing, greatly diminishing the acuteness of the numerous angles formed by the convolutions of the small intestines, and which would otherwise present so many serious obstacles to the progress of the solid and fluid parts of the mass. Having been conveyed by these means to the extreme termination of the ileum, the contents, now reduced to excremental matter and a considerable quantity of a peculiar fetid gas, are propelled into the cæcum through the ileo-cæcal valve, which is so constructed that, at the same time that it affords them a perfectly free passage, it effectually prevents even their fluid or gaseous portion from returning, either in health or disease, into the ileum. Having entered the cæcum, they are there very differently circumstanced, and moved forward by a very different process. But as I find it most difficult, if not impracticable, to explain these points in a sufficiently clear and satisfactory manner, without first disposing of others, I shall defer their consideration for the present, and assume that the fecal mass has been conveyed into the sigmoid flexure, and that the repeated descent of similar masses causes this portion of the colon to become distended, and to ascend from the cavity of the pelvis into the left iliac fossa. When this occurs, the flexure, in proportion to the rapidity and degree of its distension, begins to turn upon the contracted rectum as upon a fixed point, until at length, like the stomach, it directs its greater arch forwards and upwards, and its lesser backwards and downwards. By this movement the contents are brought somewhat perpendicular to, and so as to bear directly upon the upper extremity or annulus of the contracted rectum, but as their mere weight is insufficient to force a passage downwards, and as this end cannot be accomplished either by such gentle pressure as that exerted by the alternate contraction of the diaphragm and abdominal muscles in ordinary respiration, or by the efforts of the flexure itself, in consequence of its muscular power being so very inferior to that of the rectum, they are compelled to remain stationary, until such time as the increasing accumulation and distension produce a sense of uneasiness sufficient to call into action those great expulsive agents, the diaphragm and abdominal muscles. These muscles, instead of acting alternately, now act simultaneously, compress the abdomen and its contents on all sides, urge the free and floating mass of small intestines downwards, and even into the cavity of the pelvis, so as to press forcibly upon not only the distended sigmoid flexure, but also upon the cæcum and the urinary bladder. By these means the contents of the distended flexure are acted upon in every direction, and so as to be impelled against the upper extremity or annulus of the contracted rectum with a force sufficient to compel the parietes of this intestine to separate and afford a free passage. The nisus now ceases, but as soon as the rectum becomes filled, it is roused to make an expulsive effort, by which the whole of its contents are driven and impacted into the pouch. Here their accumulation produces a great sense of weight and uneasiness in the perineum, an urgent desire to go to stool, and a still stronger nisus, by which the sphincters are forced open and dilated, and the final expulsion of the egesta is effected. But the urinary bladder, although it is subjected to considerable pressure during this process, is not evacuated at the same moment, but immediately after, because, during this the last stage of the process of defecation, the accumulation within the pouch and dilated sphincters presses up the prostate gland against the arch of the os pubis, and thus effectually prevents the flow of urine, until the accumulation is removed. The evacuation of the rectum and bladder being completed, immediately the nisus ceases, the rectum and the sphincters return to their former state of contraction, the diaphragm reascends, carrying with it and restoring to their proper situations the liver, the stomach, the spleen, the small intestines, the cæcum, and the ascending, transverse and descending portions of the colon. But the inferior portion of the sigmoid flexure is differently situated. Having a remarkably long and free process of peritoneum, and being empty, it is compelled, during the last expulsive nisus, to occupy part of the space which the evacuation of the bladder and rectum leaves in the cavity of the pelvis, and must of necessity remain in this situation, until it becomes again distended; because, as a mere glance will show, the manner in which the peritoneum connects the small and large intestines with the diaphragm is such, that from the descending portion of the colon being bound down to the abdominal parietes, this is the only portion of the intestinal canal which does not follow, and is not in the least influenced by the action of the diaphragm. This is the fact which induced me to assume that the situation of the empty flexure in the living body, is the same as that in which it is uniformly found after death." 23.

The passage of the excrementitious matter from the cæcum to the sigmoid flexure is the next subject of demonstration, and we suspect that our readers will not consider the author quite so happy in his explanation of this process. We shall therefore again let Dr. O'B. speak for himself.

"The excrementitious matter passing from the ileum into the cæcum, becomes accumulated in the latter intestine, and prevented from being moved upwards, from a variety of causes, among which may be mentioned the very acute angle at which the ileum enters the cæcum; the greater size and capacity of the cæcum compared with the ileum which enters, or the ascending colon which leads from it; the long course which the ascending colon takes against gravity; and the absolute necessity of the cæcum becoming filled before it can either be excited to or supported in any expulsive effort. But there is obviously another and a still more powerful cause than any of these for accumulation in this situation. *It is this, while the solid and fluid portions of the excrementitious matter are filling the cæcum, the gaseous portion, not being subject to the same laws and disadvantages, ascends into and ultimately fills to distension all that space intervening between the cæcum and the sigmoid flexure, and by the pressure which it exerts, as effectually prevents the ascent of the contents of the former intestine as the introduction of air into the tube of a barometer prevents the ascent of the mercury.*

Although thus difficultly and peculiarly situated, the contents of the cæcum are transferred to the sigmoid flexure by a very simple process, which is this. From the above causes, the accumulation of flatus in the colon increases, until at length the great expulsive agents are called into action, and the bowels evacuated in the manner just described. One of the great impediments, the flatus, is thus removed, but at the same instant of time that this is effected, the diaphragm and abdominal muscles are not only compressing the cæcum in the way already mentioned, but also propelling the contents of the small intestines into the already distended cæcum in considerable quantity, and with a force at once sufficient to excite this intestine to an expulsive effort, and compel its contents to ascend and occupy the vacant space above it; and once put thus in motion, the mass is easily forwarded by the action of the diaphragm and abdominal muscles, aided by that of the intestine itself, into the descending colon, from whence its descent into the flexure is comparatively easy and obvious. As a proof that this is rarely the process by which the cæcum is unloaded, and the flexure filled, and that it is one which is not slowly, but very quickly, completed, I shall now state a few facts with which the nature of my examinations into the state of the rectum in healthy persons has made me acquainted. In the course of these examinations, it often occurred that on passing the gum elastic tube into the flexure, the escape of flatus, but not of fluid feces, the unresisting feel communicated to the hand, and the unsoiled appearance of the instrument, when withdrawn, satisfied me that no feces were then in the flexure; yet, on reintroducing the instrument in four or five minutes after, I almost uniformly found that more or less fluid feces passed through it, that its upper extremity was coated with solid feces on being removed, and that the person discharged a solid stool soon after.

In arriving at this stage of the subject, evidence has been adduced which appears to bear me out in concluding, first, that the cæcum is considerably distended before it is unloaded; secondly, that the whole of the mass by which it is distended, and no more, is transferred at each time that it is unloaded; thirdly, that at the moment of going to stool, there is generally one mass of fecal matter in the cæcum and another in the sigmoid flexure, and consequently that these may be considered as the measure of the quantity discharged when the bowels are said to be freed; *fourthly, that as two distinct acts of expulsion are always required before a healthy person finds his bowels sufficiently freed, the capacity of the cæcum may be received as the measure of that of the rectum.*"

The compression and detention of the fæces in the cæcum by the confined gases in the arch of the colon, is certainly a novel idea, if not an ingenious one; but we apprehend that the extrication of the gases from this neutral ground is not confined entirely to the time of defecation, or else our auditory and olfactory nerves have often deceived us. That the contents of the caput coli only move forward to the sigmoid flexure, when the latter is emptied into the rectum, thus forming the second edition of defecation, we will not positively deny, though we candidly confess that we are not prepared to admit its correctness. The fact, if constant, of there being two or even three distinct portions of fæces evacuated at each period, would not prove this position, since the same phenomena might be expected from two or three different expulsive efforts of the rectum and sigmoid flexure alone. The head and the body of a child are not usually expelled by a single effort of the uterus and abdominal muscles. Moreover, in healthy and natural defecation, the whole consists, very frequently, of one long and connected cordon of solid fæces. Whoever has had the curiosity to attend to this rather unsavoury, but philosophical subject, on the banks of the Ganges or other streams in India, or on the gangways of a ship, on a long voyage, while contemplating the blue waters rushing along the sides of the vessel, must have had numerous opportunities of confirming this last observation. Again, those who have attended to this subject, must have had ample demonstration, during the operation of purgatives or lavements, that the transverse arch of the colon is not always occupied by air alone. The scybala that so frequently present themselves on such occasions, shew that the cells of the colon are too often and too long inhabited by lazy and unprofitable tenants.

But Dr. O'Beirne has placed himself, we think, in a very awkward dilemma. He tells us that the contents of the cæcum are kept from ascending up and along the colon by the confined gases, till the contents of the sigmoid flexure are evacuated, when the gas escapes, and *then* the cæcum is evacuated, and its contents sent forward and evacuated also by the expulsive efforts. Now he has not shewn us how the sigmoid flexure of the colon gets its *first* cargo of contents every day!! We cannot conceive how the author can extricate himself from this embarrassment unless he maintains that the contents of the cæcum, when transferred to the sigmoid flexure, remain there till next evacuation. This cannot be his meaning, according to the passage marked in Italics, respecting the "two distinct acts of expulsion," at each complete process of defecation.

It might not be very difficult to maintain that the cæcum is a kind of cess-pool, whose contents daily overflow into the transverse arch of the colon, where there is a series of smaller cess-pools (the cells of the colon) still farther to delay the fæces for the extraction of any nutritious particles, till at length the sigmoid flexure of the colon, and the rectum became charged and stimulated to expel their contents.

From physiology our author naturally directs his attention to pathology, the knowledge of which is essential to the success of therapeutics. He justly observes that irritation in the digestive tube itself—in either of its nervous centres—or in organs with which the tube sympathises, may be so mild as merely to hasten the process of defecation, in the form of slight diarrhœa—and if in a higher degree, the effect will be felt most in that part of the tube which is most muscular and excitable. Such effect he thinks will chiefly appear in the rectum, already in a state of contraction, and now having its contractile force augmented—hence constipation of the bowels more or less in degree.

"If the constipation proves obstinate, the patient feels perhaps no inconvenience, and continues to indulge his appetite as usual, until, at length, the cæcum and colon become so distended, that they can no longer admit the contents of the ileum, and then pain in the bowels, severe twisting round the umbilicus, vomiting, and, in short, the symptoms of colic ensue. If this state be suffered to continue for a certain length of time, the solid, fluid, and gaseous contents soon cease to find an entrance into the colon, accumulate in the ileum and other small intestines, rouse these intestines and also the abdominal muscles into strong action, and thus finally become the cause of their own expulsion by the mouth, the only direction in which they can pass, or encounter least resistance. In this way, and without in any manner recurring to the gratuitous assumption of an inverted or antiperistaltic motion taking place, stercoraceous vomiting is superadded to the other symptoms, and colic is converted into ileus or ileac passion. Lastly, if the patient be not relieved from this state, he will either die, exhausted by excessive pain and debility, or the following series of effects will be produced: the distension of the whole of the intestinal canal goes on increasing, until the laminæ of the mesentery become forcibly separated just as they go to invest the intestines, and the sub-serous tissue is either unnaturally stretched or torn; this tissue soon becomes the seat of inflammatory action, and thus, according as this action may extend itself along the mesentery, or confine itself to the serous coat of the intestines, will ileus be converted into either peritonitis or enteritis." 29.

The chief obstruction is considered by our author as existing at the upper part of the rectum, between the pouch and the sigmoid flexure of the colon, and this point he conceives to "be much more exposed to both mechanical and chemical irritation than any other part or point of the intestinal canal."

"Indeed, when we consider how frequently accumulations take place in the flexure from the common but pernicious habit of disobeying natural calls to stool; how commonly stimulating and improper articles of food, and the most drastic medicines are used; and how often the hepatic and intestinal secretions become, from these and a variety of other common causes, of a highly vitiated and irritating nature, it is scarcely possible not to admit that this particular part of the intestine is in a very constant state of excitement and spasm. Hence it is, that spasmodic stricture is of such frequent occurrence in this particular situation; and that constipation is so general a feature of disease. It is in this way also that the narrow neck or contraction so often observed, in subjects of all ages and of both sexes, at the extreme termination of the sigmoid flexure, is to be explained, and not by considering it the result of congenital malformation, as Mr. White of Bath, and, more recently, Mr. Salmon and Mr. Calvert of London, agree in believing. Previous to the formation of spasmodic stricture in this situation and in this way, that particular part of the mucous membrane which lines the stricture, is far more exposed than any other to the irritation arising from the weight, impulse, and perhaps acrimonious nature of the contents of the flexure; but it becomes still more and more exposed after the formation of the stricture, until at length it is excited to a mild kind of inflammatory action, which soon extends to the corresponding portion of the muscular coat, but is prevented from extending further by the effusion of coagulable lymph, and the formation of adhesions between the two coats ; thickening and, of course, coarctation of the parietes of this part of the canal follow, and thus spasmodic is converted into organic stricture." 33.

Time, he thinks, and natural predisposition concur, in giving a tendency to such a state to degenerate into a scirrhous and malignant structure. This state is accelerated, he thinks, by the pressure of accumulated masses in the sigmoid flexure of the colon, obstructing the return of blood from the hæmorrhoidal vessels.

Besides the forced state of contraction in the rectum, as a cause of costiveness, there are others, our author observes, such as strangulation or invagination of portions of the intestinal canal—the presence of intestinal calculi—collections of fruit-stones in the bowels, forming nuclei for concretions—a retroverted, scirrhous, or a gravid uterus—the formation of large tumours external to the rectum, besides many others growing on its internal surface. All these act, in a great measure, mechanically, and their removal or mitigation require various and different modes of treatment.

The colon, Dr. O'B. remarks, is rarely found to be the seat of obstruction. The contractions which we sometimes see in this gut are, he thinks, not seldom the " effect of the purging which so often occurs, either immediately before or *after* death." This is surely a forced, as well as fanciful conclusion. The following is more rational.

" But if the descending colon should happen, from any cause, to discharge into the sigmoid flexure a greater quantity of matter, and in a more rapid and sudden manner than usual, the latter, by making a sharp and nearly complete turn upon its own axis, may become so twisted as to cause a very perfect and formidable species of obstruction. It is obvious, however, that the twist so formed is not likely to occur near to the rectum or fixed point, or in the distended portion above it, and that it will take place much more frequently at the upper and less distended portion of the flexure. It is also obvious that this twist could not be produced in the first instance, nor, in the next, exist for a longer time than the expulsion of the offending matter would require, if the rectum did not remain contracted, and firmly oppose the escape of the contents of the flexure." 42.

Although the mucous membranes are much more prone to ulceration than adhesion, yet the adhesive effusion occasionally takes place in the rectum during a highly-inflamed state. But before the adhesion can acquire consistency, the contents of the colon, from above, either completely break up the effused lymph, or extend it into the form of membranous filaments, crossing each other, as chance may determine, in various directions.

" In this manner is formed that kind of filamentous network which has been occasionally found in the rectum, and which, although it permits the escape of all the fluid feces and flatus, effectually obstructs the discharge of all solid excrement, and ultimately causes a sort of constipation which requires the obstructing filaments to be divided before it can be removed. One of the most remarkable instances on record of obstruction from this cause will be found detailed by M. Renauldin, in the Dictionnaire des Sciences Medicales, article 'Constipation.' It appears that the subject of it, a medical officer of the French navy, had suffered severely from obstinate constipation since his birth and up to the forty-fourth year of his age, when it caused his death ; and that, on examining the body, the anus was found excessively dilated, the cavity of the rectum crossed by a fibrous partition situated a little above the anus, and that above this again the rectum and the other intestines were so enormously enlarged as to fill the cavities of the pelvis and abdomen, and to contain thirty kilogrammes, or more than eighty pounds, apothecaries' weight, of a blackish brown, pultaceous, and offensive matter." 44.

The pendulous position of the belly in advanced age, and the weakness of the expelling muscles, contribute also to constipation. The compaction of hardened fæces in the rectum, our author thinks, is very rare, and only observed in paralytic or very aged, infirm, and sedentary persons.

Mr. Copeland has described another cause of constipation—hypertrophy of the sphincter muscle, with inflammation, causing a most painful disease, the involuntary contractions of the sphincter being compared to the pains of labour.

"They frequently come on immediately, but more usually about an hour or more after each evacuation, and sometimes continue till the succeeding one; in some instances the complaint goes on to produce suppuration, and consequent fistula; sometimes the irritation is propagated to the neck of the bladder, and produces a retention or impediment to the urine. I have seen it, in two cases, extend up the canal, and give rise to attacks of violent colic, and an increased secretion from the whole inner membrane of the gut, so that an evacuation of mucous cylinders, of the size of the part of the canal where they are formed, or of detached pieces of mucus, are seen mixed with the feces; yet all this has been finally removed by the bougie. I have seen it followed by a constant evacuation of shreds of coagulable lymph, which has continued through life, and produced the greatest distress. When this substance accumulated in the bowels, it was accompanied with pain, which continued until it was discharged." 51.

The principal means of relief are opium and the bougie. Dr. O'B. conceives that all Mr. Copeland's cases afford evidence of stricture at the upper extremity of the rectum, and of great irritability and sensibility, not merely of the sphincters, but of the whole bowel. The bougies, in Mr. C.'s cases, were usually passed up six or seven inches. Dr. O'B. does not mean to deny the existence of spasmodic stricture of the sphincter ani, as an independent affection, since Baillie, White, Dupuytren, Boyer, Colles, and many others have settled that question; but he maintains that the recorded descriptions of such cases do not shew that they were attended by constipation.

"It appears then from all that I have said and advanced on the subject, that, with the exception of the comparatively rare instances in which alvine obstruction is the consequence, either of the cavity of the rectum being traversed by membranous filaments, impacted with indurated feces, filled by tumours and other excrescences, or obliterated by the pressure of tumours or of displaced or morbidly enlarged organs external to it, constipation is invariably an effect, and nothing more or less than an effect of an unusually contracted and impervious condition of the rectum, produced by a more than usually firm and strong action of its own powerful and highly irritable muscular parietes, and maintained by a variety of favourable circumstances already explained. And it does not appear that even that species of the affection which arises from twisting of the sigmoid flexure, forms an exception to this general rule." 54.

He now proceeds to the curative indications and treatment of constipation, confining his observations to the disease as it arises from ordinary causes.

Assuming, then, that the rectum is contracted, and the colon distended with fæces and flatus, the obvious indication, he observes, in the treatment of constipation, "is to mechanically dilate the rectum, so as to open and form a free communication with the colon, and thereby not only give exit, but circulation to the matter so confined." This plan of treatment, he avers, will be found to exceed all others in the important points of safety, certainty, and expedition.

"This plan consists, as the reader must have long since anticipated, in the introduction of a large sized gum elastic tube through the anus into the sigmoid flexure of the colon, and, after giving exit to such flatus and fluid feces as may happen to escape, adapting to it a proper syringe, and throwing up such purgative fluids as circumstances may make it necessary to select. The first instrument that I employed for the purpose of dilating the rectum, was a gum elastic catheter of the largest size; the next was the tube of the stomach pump; but I soon found that these, in consequence of having eyes at the sides, but no opening at the point of their upper extremity, caused the fluid to be expended on the sides, and not driven to a sufficient distance up the cavity of the bowel; accordingly I cut them across below the eyes, and in this state used them, having first rubbed oil into the cut surface. Of late years I have been in the habit of using an apparatus, which consists of two gum elastic tubes and a brass syringe, and may be described thus: one of the tubes is of the largest size, eighteen inches long, open at both ends, bulbous at the upper extremity, and has, at the lower, a brass ferrule made to receive and accurately fit the short pipe at the end of the syringe. The other tube is by one-third shorter, and, in all respects, the same as that attached to the horizontal pipe of the stomach pump. The syringe has, on one side, a spring lever so constructed, that, when firmly pressed upon, it turns a simple stopcock, and forms a communication between the chamber and the short tube at the extremity, while it closes that which had previously existed between the chamber and the short tube situated at one side; in short, it is Weiss's syringe, as improved by the late William Lloyd, an obscure London artist, who added the spring lever, an addition which has perfected, and greatly increased the facility of working the instrument. The manner of preparing and using this apparatus is very simple. If the tube has been kept in a warm situation, it will not be sufficiently stiff for the purpose, and will, in all probability, become doubled on itself in the act of introducing it. Whenever this occurs, it should be placed for a few minutes in cold water, and afterwards in a current of air, until it acquires the necessary degree of stiffness; its upper extremity is then to be well oiled. The syringe should be placed for a few minutes in warm water, be then removed, and well dried, afterwards have the shorter tube fixed on its horizontal pipe, and be filled with the fluid intended to be injected. In filling or charging it, either the short pipe at the end of the instrument, or the extremity of the short gum elastic tube may be immersed in the fluid; but whichsoever we may happen to use should be steadily kept beneath the surface, in order to avoid the inconvenience of drawing in air. With the same view, also, it will be better to draw up the piston slowly and evenly, than in a rough and rapid manner. The tube and syringe being thus prepared, an assistant is to be directed to hold the basin containing the remainder of the fluid to be injected, and when the syringe is to be recharged, to keep the extremity of the smaller gum elastic tube beneath the surface of the fluid; a chamber-pot is to be at hand to receive any fluid feces that may escape through the tube, and, according to the circumstances of the case, either a close-stool or a bed-pan is to be prepared and ready for immediate use. Having made these arrangements, all of which will be found very useful in their way, the patient is to be turned on his left side, directed to draw up his knees, and the point of the tube, directed by the fore-finger of the right hand, is to be inserted into the anus, which is often so tightly constricted as to make it a matter of some difficulty and requiring some force to effect its insertion. This being accomplished, the instrument is to be directed and firmly pressed upwards, inch by inch, and as nearly as possible in the course of the intestine. If the expulsive efforts be violent, which will occasionally happen, it will be advisable to yield somewhat to them, and take advantage of their intermissions to pass it higher and higher. When it has reached, in this way, the height of eight or nine inches, the opposition to its further passage will be found considerably increased; but instead of yielding to it, the pressure upward must be gradually increased, until such time as the resistance is completely overcome. The moment that this occurs, the point of the instrument passes rapidly onwards, and as if through a very narrow ring, and the escape of either flatus or fluid feces, or of both, takes place, gives immediate and greater or less relief to the patient, and assures the operator that the upper extremity of the tube has entered the sigmoid flexure. But if neither flatus nor fluid feces should happen to escape, at this time, he may be assured that the instrument is blocked up with and embedded in a mass of solid feces. Indeed, the subsequent steps of the operation will remove all doubt of its having been so circumstanced, for, in all such cases there is greater difficulty than usual experienced in discharging the syringe, and the tube on being removed, will be found to have its cavity blocked up, and several inches of its surface coated with feces. The next step is to insert the short pipe at the extremity of the syringe into the ferrule at the lower end of the tube, and by pressing firmly upon the spring lever with the left thumb, and then depressing the piston, to discharge the contents of the syringe into the colon. The degree of force necessary to depress the piston will be found to be moderate, except in the cases just noticed, where the tube is plugged up with solid feces, and embedded in a mass of the same; but even in these, the resistance soon gives way before the impetus of the injected fluid. As soon as the syringe is discharged, the thumb is to be removed from the lever, the point of the shorter tube to be turned into and immersed in the remainder of the purgative fluid contained in the basin, the piston drawn up, the syringe filled, then discharged as before, and so on until all that remains of the fluid is injected. After the first discharge, the patient will express a desire to go to stool, and be very urgent to be allowed to do so after every succeeding one; but the effect will be rendered much more complete by not complying with his entreaties, and persevering until the necessary quantity is thrown up, when the tube is to be slowly withdrawn. The moment that this is done, he will rarely fail to hurry to the night-chair, and discharge a copious and, in many instances, an enormous stool." 64.

Few of our readers, we imagine, will have perused this long detail of operations, without having repeatedly asked themselves the question—is this then the remedy for constipation of the bowels? The operation, in every instance, must require one able surgeon and an assistant! How is this practice ever to become general? How many patients labouring under constipation of the bowels (especially females, who are the most numerous) will submit to it? Not one in one hundred! It is clear that the remedy is only adapted to dangerous cases, where the common means fail, and where the life of the patient is in danger; or, to a few obstinate cases in men, who may be inclined to submit to much trouble and inconvenience to get rid of an obstinate evil.

Dr. O'B. informs us that he has now employed this mode of treatment for nine years, and with decided and unexampled success. This we doubt not; but still we think the practice can never become general, because it cannot be put in execution by the patient. Dr. O'B. next proceeds to the narration of cases in support of his doctrine and practice, some of which we shall condense, as they are far from being devoid of interest.

*Case* 1. This was a lady about 40 years of age, subject to gout. Our author found her, in February, 1824, with both knees swelled, red, and painful, preceded by nausea, eructation, and loss of appetite. These symptoms were abated in two days, by antiphlogistics and colchicum. The bowels were opened, and the medicines continued. On the fourth morning she awoke with intense pain in the region of the stomach, followed by incessant vomiting. The gout had retroceded from the joints, and she was cold in the extremities, with sunken countenance, quick, weak pulse, and tenderness in the epigastrium. Bowels not moved since the preceding evening—"in short, the case was evidently an example of metastasis of gout to the stomach, and the patient's life appeared to be in imminent danger." Dr. O'B. endeavoured by enemata, and various other means, to evacuate the bowels, and assuage the pain and irritability of the stomach—but all in vain, though he had the able assistance of Dr. Crampton. As a last effort, a large enema, composed of scammony, jalap, colocynth, &c. was attempted to be thrown up by the nurse; but much force was necessary, and the contents returned as fast as thrown up. Next morning a tube was introduced, and passed up into the sigmoid flexure, as described in the text. A limpid serous fluid flowed rapidly from the tube till three imperial pints or more were discharged. The patient experienced sudden and decided relief. During the remainder of that day and the next, the same kind of fluid continued to be discharged, unmixed with any feculence. She was quickly restored to health.

*Remarks.* This case is fairly considered by our author as a metastasis of gout to the mucous membranes of the primæ viæ, producing such a serous secretion as to make it somewhat resemble cholera. We think that few observant practitioners will doubt that the immediate cause of this retrocession of gout to the stomach and bowels, was the colchicum.

*Case* 2. This is headed "Spinal Irritation," and the subject of it was a young lady who, from the pressure of tight stays, became affected with pains in the back, shooting round to the stomach and other parts, aggravated by food. This induced her often to fast for 12 hours, and then eat extremely little. The bowels became very confined, and she frequently vomited up what she ate. In December, 1830, the constipation began to resist enemata and other means, and to produce great distress. In May, 1831, she came under Dr. O'B.'s care, and affirmed that she had passed neither fæces nor flatus for six months, notwithstanding the various means that were employed.

"At this period, her state, in other respects, I found to be as follows: she complained of total want of sleep, and the impossibility of procuring it by artificial means. The irritability of her stomach was such, that the only sustenance she could take, or had taken for two months previously, consisted of a tablespoonful of milk and lime-water taken frequently in the day, but vomited up nearly as soon as it was swallowed, apparently little altered in quality or diminished in quantity. She was very weak, but could walk about with assistance, and though thin, not as emaciated as might be expected. No unusual fulness or tenderness on pressure was perceptible in any part of the abdomen, her pulse was weak but regular, her tongue covered with a cream-coloured fur, her menstruation, as throughout her illness, regular as to periods, quantity, and quality, but attended with severe pain." 78.

From the success which our author had obtained on various other occasions, he confidently promised to remove, not only the constipation, but all the other symptoms. In the latter, however, he failed. The gum elastic tube having been introduced, and nothing coming away, it was withdrawn, and its extremity was found to be covered with solid fæces.

" It being now clear that the sigmoid flexure contained a mass of solid excrement, the tube was again introduced, and in doing so the same difficulty was experienced, and it became necessary to use the same degree of force. Still no flatus passed off. The syringe was now adapted to the tube, and the whole of the injection thrown up. While this was doing, she became very urgent to be allowed to go to the night-chair, but her entreaties were not complied with, until the whole of the fluid had been injected. The tube was then removed, and, in less than two minutes, she passed one of the most enormous stools I have ever seen ; it nearly filled a large-sized chamber-pot, was altogether solid, perfectly natural looking, and arranged in remarkably thick coils. Soon after, she expressed herself as being greatly relieved from the spasms which she had so long felt in the stomach and bowels, but complained of feeling a great degree of weakness." 80.

Although considerable quantities of fæces came away next day and afterwards, she continued to pass sleepless nights, and to be harassed with vomiting and spasms—milk, in spoonfuls, being the only nutriment she could take. In short, her situation was worse than when our author first saw her. At length it was discovered that there was a tender portion of spine, from the sixth dorsal vertebra downwards. Leeches were immediately applied, and then blisters, with sensible relief. The tartar-emetic ointment was then rubbed along the spine, and strong pustulation produced. From that time the stomach became retentive—the rectum gradually ceased to offer resistance to the tube—and the necessity for introducing it occurred less frequently. Sleep returned by degrees, as did strength. She is now in robust health.

*Remarks.* This case is certainly very interesting and instructive.

" Morbid irritation conveyed directly from the spinal marrow, through the medium of one of its offsets, the hæmorrhoidal nerve, to the rectum, caused that intestine to contract in a powerful manner, and in this way produced such an accumulation of feces in the sigmoid flexure of the colon, as would, no doubt, have ultimately produced ileus or colic, or perhaps enteritis, if the state of the patient's stomach had not incapacitated her from taking any greater quantity, or any other kind of food. That such was the cause of the singularly obstinate constipation which prevailed, and the precise mode in which it was produced, is obvious from the different states in which the rectum was found before and after the discovery and removal of the irritative condition of the spinal cord ; and as to the other phenomena of the case, the irritability of the stomach and spasms of the abdomen, they are at once explained by the intimate nervous connexions which the spinal cord is known to have with the parts so affected." 83.

Case 3.—*Mania.* This case was communicated to our author by Dr. M'Dowel, one of the surgeons of the Richmond Surgical Hospital. A young lady, whose catamenia had become suppressed, attempted to cut her throat on the 21st of February, 1830. The bowels being constipated, purgatives were ordered, and acted freely. She remained sullen for several days, and then became violent, with constipated bowels. Calomel and colocynth failed to act, as did castor oil and purgative draughts. Enemata, exhibited in the usual way, failed also. After seven days of obstinate constipation, the tube of the stomach-pump was passed up into the sigmoid flexure of the colon, when a loud burst of flatus, followed by large discharges of liquid fæces, took place, with decided and general relief. From this period she recovered rapidly and perfectly.

Case 4.—*Narcotic Poisoning.* To a boy, 6 years of age, a cup of what was considered to be senna tea, was given to open his bowels. He immediately cried out that he had got the cholera—became delirious, convulsed, and attempted to bite himself and others. A similar dose had been given to one of his sisters, followed by similar symptoms, which ceased on full vomiting taking place. It was now ascertained that, instead of senna, the herb and seeds of stramonium had been exhibited. An emetic was therefore quickly given to the boy, producing copious vomiting; but without any sensible relief. The next object was to free the bowels, and fortunately our author had with him his improved self-injecting apparatus. The tube being introduced, and a purgative enema having been thrown up, " a great quantity of flatus and of solid dark green fæces was discharged." This measure, with some others of a general nature, produced decided and permanent relief.

*Remarks.* Although this case is interesting in itself, we do not think it proves much in the question agitated by the author. There is no proof that an enema exhibited in the usual way would not have been equally successful.

Case 5.—*Abdominal Tumours.* An aged gentleman, accustomed to hunting and field-sports, gradually lost his appetite, became costive, flatulent, and unusually prominent in the abdomen. At length, a tumour appeared in the left side, which increased in size and became painful. Upon careful examination, our author came to the conclusion that it consisted of indurated fæces. He accordingly introduced the tube, and threw up a turpentine enema. Some flatus and hardened balls of fæces came away. Purgatives were then exhibited by the mouth : but no relief was obtained. Another medical gentleman now joined in consultation, and disagreed with our author respecting the nature of the tumour, and recommended external friction, in addition to purgation. This plan also failed, and surgeon Crampton was added to the list of consultants. He agreed with Dr. O'Beirne, and croton oil was exhibited, with other purgatives. By steady perseverance, several scybala were dislodged daily, not much larger than peas, the tumour gradually lessening in size, and eventually disappearing on the evacuation of a large lump of excrement, followed by a quantity of fluid fæces. He was much relieved—treated afterwards by purgatives and tonics, and returned home in tolerable health soon after.

*Remarks.* We have been often deceived by these congregated masses of hardened fæces in the colon, and have taken them for organized tumours of a much more formidable character. The same mistake is made by others, and we have no doubt that cures are often attributed to men and measures, where little real credit is due to one or the other.

CASE 6. This was a young gentleman who had been long in bad health, and whose digestive organs were in a highly disordered condition. The primary history is too long for detail, and we shall come at once to the period when he was taken charge of by our author. This was on the 21st Oct. 1831.

"His countenance was then anxious and sallow, his pulse regular, but weak, his tongue covered with a brown fur, his urine scanty and high-coloured, and stained the chamber-pot of a delicate pink colour. He had had no discharge of any kind from his bowels for forty-eight hours, and, for several days before, he regularly vomited up every thing he ate or drank in about twelve hours after it had been swallowed. On examining the abdomen, which was found to be greatly swelled and tympanitic, yet not in the least painful on pressure, he directed my attention to a tumour of the size and shape of an orange, situated in the left iliac region, near to the anterior superior spinous process of the ilium, and extending above and below this process. It was very moveable, firm, hard, rather unequal, free from pain, and easily embraced by the fingers and thumb. He complained of 'frequent rumbling of wind in the bowels, which he could not expel, and which seemed to be stopped at the tumour,' and also of a sense of weight and uneasiness in the lumbar regions. My first step towards his relief, was to pass the tube into the colon. This being effected, but with unusual difficulty, a considerable quantity of wind, and some fluid feces escaped, and gave him such relief that no injection was thrown up, and he was directed to take a drop of croton oil immediately, to have a large belladonna plaster applied over the abdomen, and to take, at bed-time, a diuretic draught, composed of two ounces of infusion of juniper, a drachm of nitrous æther, and a scruple of acetate of potash." 100.

For two or three days he seemed rather better, but on the 25th he was worse than ever. The tube was again passed with difficulty. A quart of warm water was thrown up and retained. Suddenly the patient raised himself in bed, and seemed to be dying, from some violent internal struggle. In a few minutes, the stomach discharged more than three quarts of semi-fluid feculent matter. When the vomiting ceased the pulse rose—he took some brandy—and had a tolerable night. He expired the next day, during a fit of vomiting.

The following were the appearances on dissection.

"The stomach, duodenum, jejunum, and the two upper thirds of the ileum, enormously distended with fluid feces, but no morbid alteration of their coats, except at a point to be mentioned hereafter. The lower third of the ileum, the cæcum, the whole of the colon, and the rectum, as far as its pouch, contracted to the greatest possible degree, but also free from any morbid condition of their coats. A large tumour, situated in the upper part of the left iliac fossa, and formed by a remarkably thick layer of firmly organized coagulable lymph enveloping, first, a turn of the ileum greatly thickened in its parietes, for nearly the extent of two inches above the commencement of its inferior third; secondly, about two inches of the sigmoid flexure in a highly contracted but perfectly sound state. In the interior of this turn of the ileum, and on that side of it next to the sigmoid flexure, a circular opening lined with a dark red fungous membrane, large enough to admit the thumb, and leading into a kind of cavity situated between the ileum and sigmoid flexure; and the passage from the upper portion into the lower third of the ileum so narrow as scarcely to admit a good-sized quill. All the other viscera of the abdomen and pelvis perfectly sound." 103.

Our author next makes some observations on strangulated hernia. With few exceptions, he conceives that "strangulation is always caused by the prolapsed portion of intestine becoming so distended, generally by the gaseous, and very rarely by the fluid or solid contents, as to be pressed forcibly against the margins of the opening or ring, and to be no longer capable of re-passing through it, and returning into its natural situation in the cavity of the abdomen." After passing some strictures on the application of ice—bleeding—and tobacco enemata, our author proposes his own remedy.

"The question, therefore, proposes itself, is there any other more certain mode of accomplishing this object, that has not been either proposed or tried, and that is, at the same time, free from all objection? It appears to me that there is. It is this. To introduce a gum elastic tube into the colon, and to leave it there until the large intestines, and eventually the hernial tumour are emptied of their gaseous contents. If the bowels happened to be well freed, or to contain but a small portion of solid feces, at the time the strangulation took place, success might reasonably be expected from this mode of proceeding; and if they happened to be loaded with solid matter at that time, it would only be necessary to introduce the tube more frequently, and at intervals of a few minutes between each introduction, first, to empty the sigmoid flexure; secondly, the cæcum; thirdly, the hernial tumour, in order to effect the object in view." 113.

As it is but recently that Dr. O'B. adopted this idea, the facts which he is able to offer are not numerous. As the subject is important, we shall condense the greater number of these into as small a compass as possible.

*Case* 8. A female, 20 years of age, was admitted into the Jervis Street Infirmary, 16th August, 1831, with the following symptoms.

"On the right side, immediately below the pubal attachment of Poupart's ligament, and slightly ascending over this ligament, she has a tumour which is as large as a walnut, tense and painful, yet not discoloured. It imparts all the feel of an entero-epiplocele, and is attended with the following symptoms, viz. anxious and painful expression of countenance, jactitation, nausea, vomiting, constipation of four days' duration, pain on pressure of the abdomen, particularly over its umbilical and hypogastric regions, tongue brown at its base and along its centre, great thirst and heat of skin, pulse 100, hard and contracted. She states that at 9 o'clock on Sunday morning the 14th, she had been pumping water to supply her master's house; that at 2 o'clock in the afternoon, she was seized with sickness of stomach and violent pain in the belly; that she vomited repeatedly during the evening, retired early to bed, and there for the first time, discovered a tumour in her groin, and pain and difficulty in extending the right thigh and leg. She adds, that she went the following morning to the next apothecary, who gave her some pills and an injection, but without affording any relief. The apothecary she mentions has had the humanity to attend her to the hospital, and this gentleman states, that, suspecting the nature of the case, he gave her pills of calomel and cathartic extract, and a tobacco enema, and that he could not be deceived in asserting that he saw her vomit stercoraceous matter. It does not appear, however, that she has had what could be considered as fecal vomiting since her admission into hospital." 116.

She was bled ad deliquium, had tobacco enemata, and the taxis was employed for some time without effect. The tube was then passed into the colon, when a considerable quantity of flatus escaped, with great diminution of the hernial tumour, which now appeared to be chiefly omental. Nausea and vomiting ceased—the pain was inconsiderable—and a mild enema was administered by the tube. In half an hour the tube was re-introduced, and then came away a pint of fœtid fluid fæces. Repetitions of the enema with cathartics by the mouth completed the cure.

Dr. O'B. quotes a case from Mr. Copeland's work on the Rectum, in which a strangulated hernia was quickly reduced by the operation of dilating the rectum for the removal of a stricture. We shall quote the case.

*Case* 9. "A lady, about 40 years of age, who had been affected with an umbilical hernia for many years, was seized with violent pain in the abdomen and vomiting, and had not had any evacuation from the bowels for seven days. The rupture was painful to the touch, was of the size of a very large orange, and had been incapable of reduction for twenty-four hours. Her pulse was quick and weak; she had been taking large doses of calomel and other strong purgative medicines, without effect. In this state Mr. Ford was called to her, and I saw her with him; the rupture could not be returned by any effort that was thought prudent, and the vomiting, together with hiccough, was increasing in severity; she was bled, and directed to take some pills, with calomel and extract colocynth; and an injection of the tobacco fume was, with considerable difficulty, thrown up the rectum. It was proposed, that if these means failed of giving her relief, the operation to return the hernia should be performed without further delay.

She now happened to tell us, that she had been for many years of so costive a habit of body, that she could never pass her stools without great pain and difficulty, and seldom without the assistance of glysters, and that they were always very small in size.

These circumstances led to a suspicion that the seat of the disease was not in the hernial sac, but in the rectum; and, on passing the finger to examine the gut a firm indurated stricture was discovered about two inches up the intestine, which would not admit the point of the finger to pass it.

*A rectum bougie, of a small size, was introduced high up the gut, and retained there about ten minutes. Soon after it was withdrawn, there was a copious evacuation of the fæces, the vomiting ceased, and the rupture soon returned spontaneously; in short, all her complaints disappeared, and she was in the same state as before the attack.* By persevering in the use of the bougie, the stricture gradually enlarged, and in a fortnight she could pass her stools better than she had done for many years: she continued, however, daily to pass the bougie for about a month, and then used it only occasionally. This is now seven years ago; and I saw her very lately for another complaint, when she informed me, that she remained perfectly well of the stricture; but from fear of a return of her disease, rather than from necessity, she now and then passed the bougie, for a short time, and withdrew it again." 126.

The reader has now before him all the information on the subject which has come within his reach, and he hopes that the facts and reasonings will be sufficient to induce him to give the plan a full and fair trial—also that he will not condemn it, should it fail in those cases where the neck of the sac is found to form the stricture—where the strangulated intestine is adherent to the sac, or filled with indurated fæces—or where the case is merely one of epiplocele.

"The proposed plan can do no injury whatever, causes little or no delay to the employment of other means, and may be tried immediately after a reasonable attempt at the taxis has been made, and has failed. Should the first introduction of the tube not give exit to either flatus or fluid feces, and should the peculiar feel communicated, as well as the previous history, lead us to suspect the presence of a solid mass of feces in the sigmoid flexure, the enema catharticum, with the addition of an ounce or two ounces of castor oil, should be thrown up, in order to bring away this mass. If after the bowels have been moved, no favourable change takes place in the hernial tumour, the tube should be again introduced, and if the same happens, the same process should be gone through, and repeated until the bowels are completely freed. Once this object has been accomplished, it is to be presumed that another introduction of the tube will either enable the intestine to return proprio motu, or place it in a situation to be returned by the taxis." 128.

He does not recommend the tobacco enema on these occasions, as he thinks it does more harm than good, by paralyzing the muscles of expulsion.

Case 10.—*Colic.* We shall give this case in the author's own words, as it is short.

"Late in the evening of the 6th of Sept., 1826, the servant of Mr. W. K., of Mecklenburgh-street, called to request my immediate attendance on his master, who was, he said, 'dying from cramps in his stomach.' I found the gentleman sitting up in bed, doubled forwards, and complaining of the most acute pain in the epigastric region; his countenance was pale, contracted, and expressive of great agony, and his pulse scarcely to be felt. On inquiry, I found that his bowels had not been moved for many days; that he had been subject for years to attacks of what he called cramps in the stomach; that brandy, laudanum, æther, and other such remedies, had always relieved him; but that, on this occasion, he had had recourse to them without experiencing the slightest relief. Having brought the tube with me, I proceeded at once to pass it up the rectum. The obstruction to its passage was inconsiderable until it reached the height of eight or nine inches, when it became necessary to press up the instrument much more firmly, in order to make it enter the colon. The moment that this was effected, a burst of flatus took place through the tube: the patient instantly exclaimed, 'I am quite relieved,' and both his look and manner declared that this was the case. No injection being at hand, the tube was withdrawn, he was directed to take a purgative draught, and to have a fetid enema administered as soon as possible. Soon after, his bowels were freed, and he was perfectly well the next day. From that period to the present, I have ascertained that he has not had any return of the cramps, although, according to his own account, he had previously been accustomed to have two or three attacks of them every year." 132.

A case of enteritis is next related, in which the tube gave relief after several other and powerful means had failed. Some other cases of the same are communicated by friends.

Case 11.—*Puerperal Fever.* The following case was communicated to our author by Mr. Gregory, Master of the Coombe Lying-in Hospital.

"Johanna Barnard, aged 25, was carried to the Coombe Lying-in Hospital, on the 5th of March, 1828, labouring under such severe pains in the groins, and all over the abdomen, especially about the umbilical and hypogastric regions, as to be unable to bear the slightest pressure. Pulse small and difficult to be felt; countenance extremely anxious; great prostration of strength; bowels confined, great thirst, &c. States herself to have miscarried a few days before, in the fourth month of pregnancy, and says that she was previously strong and healthy. Twenty ounces of blood were taken immediately from the arm; an enema of turpentine; abdomen to be constantly fomented; three grains of calomel and a quarter of a grain of opium to be given every hour; enema to be repeated in three hours, and if no stool takes place, to have a castor oil and turpentine draught.

6th. Feels somewhat easier. Has had no stool. Pills, enema, and draughts to be continued.

7th. Passed a restless night, had one or two scanty motions; mouth sore, and in great pain; passed the œsophagus tube nearly its whole length up the anus, and through it injected five or six pints of warm water, some oil, and about a wine glassful of turpentine. In a few minutes after withdrawing the tube, an enormous quantity of feces came away, giving immediate and decided relief." 144.

Other cases of similar tendency are communicated by Mr. O'Hara, resident accoucheur of the same establishment.

### DYSENTERY.

Dr. O'Beirne has dedicated a considerable space to this disease, tracing the opinions of physicians respecting it from Hippocrates downwards. To these worn-out subjects we need not allude. All are now agreed that, when dysentery proves fatal we find inflammation and ulceration in some portion of the intestines. The ulceration is, of course, the consequence of inflammation—and the inflammation itself is but a secondary link in the chain of causation, there being first an increase of irritability in the lining membrane of the bowels, followed by an afflux of blood to that surface, and a great augmentation of secretion. We agree with Dr. O' B. that a very general opinion prevails, that dysentery is an inflammation of the lining membrane of large intestines only. But the opinion is unfounded; for inflammation, and even ulceration, are very frequently, if not always, found in the ileum, especially about its lower portion. The following rationale of dysentery is not new, as it is taken almost entirely from the writings of modern authors, who have treated of the disease in hot climates.

" When, as frequently occurs in autumn and the latter end of summer, a person becomes first heated, and then exposed to cold, or, which is still more frequent and influential, to cold and moisture combined, the temperature of the surface of the body is considerably cooled down, the flow of blood to, and the secretion from this surface are checked, and the consequence is, that an unusual quantity of blood is determined to the interior of the body. But this blood is not determined to any other tissue than the mucous, because, as it would appear, this tissue is but a modified continuation of, and, of all others, the most closely allied, in structure, function, and sympathy, to the skin, for which the refluent blood was originally destined. Again, this blood is not determined to the mucous membranes of the mouth, nose, fauces, pharynx, lungs, or genito-urinary organs, but to those of the liver, stomach, and small and large intestines, which are obviously infinitely more exposed to, and perhaps at the time actually labouring under, derangements which render them less capable of resisting, and more prone to receiving the tide of blood thus diverted from its natural direction. This is the congestive stage, and it marks the rigor, paleness, inappetancy, eructation, and nausea, with which the attack generally commences. The presence of such an unusual quantity of blood in these organs, destroys the equilibrium which previously existed between their vascular and nervous systems, and eventually the latter becomes excited proportionally with the former. This is the irritative stage, which so shortly precedes the inflammatory, and is only marked perhaps by the irregular and wandering pains complained of before those termed 'tormina' set in. During both these stages, there is, most likely, little or no effusion of blood, and the hepatic and intestinal secretions are arrested; but the third stage is quickly developed, and the whole of the mucous membrane of the digestive canal, from the pyloric, if not from the cardiac, orifice of the stomach to the anus, as well as that continuation of it which lines the biliary and pancreatic ducts to their minutest and most remote ramifications, are attacked with inflammation, and become highly and morbidly sensible. From this extensive surface blood is now copiously effused, and the hepatic, intestinal, and perhaps pancreatic, secretions become increased in quantity, and highly vitiated and irritating in quality. These secretions, as well as the morbid state by which they were produced, rouse the muscular parietes of the small intestines into activity, and cause them to hurry forwards their multifarious contents,—namely, alimentary matter, blood, air, and vitiated hepatic and intestinal secretions, towards the large intestines; but in their passage through the small, they necessarily create great tormina and suffering, for the containing parts and their contents no longer bear to each other the same inoffensive relation as in health, the former being much more sensible of irritation, and the latter much more irritating." 157.

The contents having passed the valve of the colon, Dr. O'B. thinks that they there become very differently circumstanced, and in a manner which requires to be clearly explained. The following is the exposition given by our author, and which he considers a tolerably accurate one.

"At the time of the attack of the disease, the rectum is, as it always is, except for a few minutes before stool, firmly and imperviously contracted; but during the irritative and inflammatory stages, it becomes, according to the principle already laid down, still more powerfully contracted; and the consequence is, that with the exception of a very small quantity of flatus, blood, and mucus, which occasionally escapes per anum, there is complete retention of the alvine contents. In fact, as far as retention of both solid and fluid feces, the bowels may be said to be as completely constipated at the early period of the disease, as they are in enteritis, for the discharges from them are then very rarely seen even tinged with fecal matter. Again, at no period of the disease is there any evidence whatever of the ileo-cæcal valve performing its office imperfectly, for if it were even true that the action of this valve may be inverted, it is certain that the stercoraceous vomiting, the supposed test of the occurrence, has never yet been observed in dysentery. Such being the state of the facts, it follows that the contents are admitted to pass freely, through the ileo-cæcal valve,—that their exit per anum is prevented by the rectum,—that their return into the small intestines is prevented by the ileo-cæcal valve, and, consequently, that once they have entered, they are are forced to remain pent up within the cæcum and colon; and that the combined effect of the peculiar structure and functions of the valve and rectum, is such as to subject these portions of the large intestines to the operation of a principle of accumulation, which is always more or less in activity. These facts have been already noticed, and are equally applicable to various other diseases, but I have considered it necessary to repeat them here, and to put them in a still more impressive shape, in order to produce as strong a conviction as possible on the subject under consideration.

Having given this explanation, I have now to revert to the early part of the inflammatory stage, in which the alimentary matter, blood, air, and vitiated secretions, have been described as being hurried on towards the ileo-cæcal valve. When all these matters enter the valve, which appears to freely admit them, they become applied to the lining membrane of the cæcum, and soon after to that of the whole of the colon; and, from having free ingress, but neither egress nor regress, become pent up and accumulated within these intestines, and subject their lining membrane, which, be it recollected, is already in an inflamed state, to a high degree of both mechanical and chemical irritation, and in fact, to an irritation which increases in intensity with every accession of matter from the superior division of the canal. In this way, the irritation and distension quickly become such as to rouse the abdominal muscles to frequent and violent expulsive efforts; but these efforts almost invariably fail, at an early period of the disease, in forcing open the upper annulus or entrance of the rectum, because this part of the intestine, by being directly exposed to the operation of the same cause by which the abdominal muscles are excited, is stimulated to oppose a degree of resistance proportioned to that of the force exerted by these muscles. Here, however, it may be objected that the fact of bloody and mucous discharges being co-existent with the inflammatory stage, is evidence of the expulsive efforts making, at least, a partial impression upon the superior annulus or entrance of the rectum; but as the lining membrane of this intestine is, at the time, in an equally inflamed state, and, consequently, as the blood and mucus discharged may be furnished from this, and not from a higher source, the objection almost ceases to be one. Indeed, it would be much better sustained, if it it could be shown that flatus was discharged at this early period of the disease; but as far as my experience of the disease goes, and it has been ample, the discharge of flatus per anum is an occurrence which is not observed for many hours or even for some days after the appearance of mucous and bloody stools. But to proceed. The inflamed lining membrane of the cæcum and colon, in consequence of being so singularly circumstanced, necessarily advances to ulceration; and it is obvious that this membrane will become much more deeply and extensively ulcerated in those situations in which the contents have been shown to become lodged, or to meet with the greatest obstruction in passing. Accordingly, pathological anatomy shews that the cæcum, and the transverse arch and sigmoid flexure of the colon are the situations in which the most numerous and the deepest ulcers are found. But if matters were to continue thus for any length of time, it is manifest that the ulcerative process would quickly destroy the muscular coat, extend to the sub-serous and serous tissues, and cause death by enteritis. In point of fact, such a conversion is far from being an uncommon termination of dysentery. Nature, however, generally makes an effort in which she succeeds in arresting the activity of this process, and causing the disease to assume a more chronic form; and the means by which she proceeds to effect these objects appear to be of a twofold description. In the first place, it will be shown hereafter that, as the disease advances, a recuperative power is bestowed upon the small intestines; that these intestines, together with the liver, no longer pour out secretions of the same vitiated and irritating quality, or in the same quantity; and that thus one great source of irritation and distension is removed. In the next place, the inflammatory action going on in the sigmoid flexure of the colon, soon extends itself, first, to the mucous, subsequently to the muscular structure of the upper annulus or orifice of the rectum; and, by thus weakening the resistance previously made by this part to the expulsive efforts, enables a quantity of flatus, blood, mucus, and occasionally fluid feces, to escape from time to time, per anum. Here is a still more effectual arrangement than the former for relieving the oppressed condition, and checking the rapidity of ulceration, of the cæcum and colon. From the upper annulus of the rectum the disorganizing process soon extends along the inferior portion of this bowel; solid and fluid feces, but exceedingly rarely what are called scybala, are found more frequently, in greater quantity, and with an admixture of purulent matter in the discharges by stool; and the disease may now be said to have assumed the chronic form." 161.

This explanation being taken for the true one, and the state of the cæcum and colon being such as our author has described it, he thinks the inference is direct, and of great practical importance—"namely, that the chief curative indication should be to pass up the gum-elastic tube, and introduce it into the sigmoid flexure, in order to give exit to the accumulated and pent up contents of the cæcum and colon." Dr. O'B. is aware of the objection that lies against this measure, on account of the highly irritable, if not inflamed state of the rectum; but assures us that the pain is momentary, and the benefit great. In consequence of the prevalence of cholera, and the disappearance of dysentery, our author had been able to use the remedy in one case only of the disease, when the body of the work was printed. This case we shall give, in order that the author may have no cause of complaint against us.

" On the evening of the 11th of April last, Miss Rosetta F., an infant nine months old, was given, while teething severely, two grains of calomel, which produced, during the night, severe vomiting, purging, and, as would appear from her cries, considerable griping and pain. The following day, and while she still had more or less of purging, the nurse imprudently carried her about in the open air, and without sufficient covering. On that night she began to pass blood and mucus per anum, unmixed with a particle of fecal matter; she moaned continually, and threw up the breast-milk, nearly as soon as she took it. To remove these symptoms, she was placed in a warm bath, had the abdomen stuped with warm water and spirits, and was given internally mint tea and castor oil, which she likewise rejected by vomiting. Various domestic injections were also administered to her by a bag and pipe, but were invariably returned unchanged, and without producing any feculent discharge; and it was observed that she passed no urine for twenty-four hours. In this state, but the general symptoms becoming daily more aggravated, she continued until Sunday the 15th, when the family feared she was dying, and I was requested to see her. Her countenance was then exceedingly pale, her feet and hands, although enveloped in warm flannel, were very cold, and she lay, evidently from great weakness, quite motionless in the nurse's lap, with her eyelids half closed, and seemingly regardless of the persons and objects about her. Her pulse was very quick and feeble, she was passing, every ten minutes, quantities of blood and mucus, untinged with fecal matter, from her bowels, and her abdomen was hard and distended. My first care was to divide the gums by a deep crucial incision made at all those points where teeth were making their way; but perceiving that she was in no degree relieved by the operation, and seeing the imminent danger in which the infant was placed, I resolved on introducing the tube. Accordingly, no time was lost in doing so, and, although it was but a size smaller than the stomach tube, no unusual difficulty was encountered in passing it, and the infant did not cry much, or seem to feel any great pain. As soon as the instrument entered the sigmoid flexure, a burst of flatus, followed by fluid feces, blood and mucus, escaped through it, and on these ceasing to come away, an injection, composed of half a pint of warm water and half an ounce of castor oil, was thrown up. The tube was then withdrawn, and immediately followed by a very considerable quantity of solid and fluid feces, mixed with blood and mucus. In less than an hour, the infant became generally warm, very lively, took the breast with avidity, and without vomiting up the milk as before; and her pulse, although still quick, became much fuller and stronger. She was now directed to have a tea-spoonful of electuary of sulphur every fourth hour, to get a little weak chicken broth from time to time, and to have the belly frequently stuped with an infusion of half an ounce of leaf tobacco in two or three quarts of boiling water, which was to be used as soon as it had sufficiently cooled down. During that day, she had two or three stools, from which blood and mucus gradually disappeared, and she slept naturally during the night. The following morning, she was so well as to require no further treatment, and she is now a healthy, strong child." 169.

Dr. O'B. does not mean to exclude other and appropriate remedies in dysentery. He thinks, however, that it will supersede the use of many other medicinal measures, and more especially blood-letting. He informs us that, about nine years ago, he published a paper "on the use and advantages of tobacco in the treatment of dysentery," and he avers that subsequent experience confirmed him in its utility. By it the necessity for bleeding was obviated—and he did not lose a single case of acute dysentery, when this remedy was employed.

"I have found it a most useful adjuvant in the treatment of colic, enteritis, and other affections of the bowels; and I have often employed it with success, in cases where, from the degree of debility which existed, general bleeding, although indicated, would have proved more fatal than the disease itself. In short, time has but served to establish the practice, and confirm all the statements made in my paper on this subject." 170.

The mode of application is a quarter of a pound of tobacco infused in four or five quarts of boiling water, and used as a fomentation, till prostration of strength ensue. Mean time a dose of castor oil is to be administered and repeated till the bowels are cleared. Here our author puts forth a pretty severe tirade against a reviewer in this Journal, who ventured to moderate Dr. O'Beirne's sanguine anticipations from having had a single case of tetanus terminate favourably after the use of tobacco. One of the passages so violently assailed is this:—"*A single case* can do no more than authorize further trials, without exciting any thing like sanguine hopes, or forming any basis for general conclusions."—*Med. Chir. Review*, 1823. It would probably be a good maxim for our author himself to observe, even on the present occasion. But, contrary to the usual disposition of critics, we are disarmed by censure, not by flattery—therefore we shall pass by Dr. O'Beirne's criticisms on the reviewer in this Journal.

The subject of tympanitis next engages our author's attention, and the tube is recommended as the remedy. Tetanus is to be the subject of a forthcoming volume, and therefore is passed over in this, with the single observation that, as obstinate constipation is a never-failing attendant on tetanus, "the introduction of the tube never fails on overcoming it."

### DELIRIUM TREMENS.

The application of the mechanical remedy now under consideration is made with a view very different from that of removing constipation. This view will be developed by the following extract.

"In almost every case of delirium tremens that I have seen, careful inquiry into its previous history has made me acquainted with a circumstance which must be familiar to every experienced practitioner,—namely, that for some time before the attack of the disease, the patient has scarcely eaten any breakfast, and very little at dinner; and has been gradually emaciating, and becoming weak and languid. This being the case, it is but reasonable to infer that the sensorial exaltation which ensues, arises as much from deficient supply of food, and, consequently, of blood to the system, as from the excitement caused by the abuse of spirituous liquors; and that one of the curative indications, and a very important one, should consist in stimulating the system naturally, as by proper nourishment, as well as by such artificial means as opium, camphor, wine, or spirituous liquors of any kind. In point of fact, remedies of the latter description must be but temporary in their effects, and act under every disadvantage, so long as the vascular system continues to be weakened by a defective supply of blood, and no longer in equilibrio with the nervous system, or capable of supplying this system with the necessary quantity or perhaps quality of blood. Accordingly, although they succeed in persons who have not had many attacks of the disease, and possess sufficient recuperative powers, we know that they too often fail in cases of an opposite description. It may be objected, however, that the patient's stomach is too irritable to bear food, or that he has an unconquerable disgust to it, and, in short, that he either cannot or will not take any nourishment, or will only take it in such small quantities as would be quite insufficient for the purpose. This objection is supported by experience and reason. But what objection can be urged against the plan of nourishing the patient per anum?" 194.

Two cases are related, in which the introduction of strong animal broths appears to have been very beneficial. We need hardly remark, however, that the common syringe would have answered the purpose just as well as the elastic tube—at least, we never find any difficulty in the introduction of broths or other enemata where they are judged necessary.

We have now given an extended and faithful analysis of Dr. O'Beirne's work—with the exception of the controversy respecting Sir Charles Bell's views of the facial nerves, and we have very few comments to make. We think it will be manifest to every one, that the new remedy for "constipation" is quite inapplicable to the general run of that complaint, under its common acceptation. Constipation of the bowels does not surely require the difficult operation of passing a tube into the sigmoid flexure of the colon every time that the bowels require evacuation—nor would this prove a remedy for constipation after all. We think it unquestionable that the tube is only a remedy for dangerous and obstinate *effects* of constipation, or other mechanical obstruction to the intestinal evacuations. In such cases, and such cases *only*, can it be applicable—and then *only* in the hands of an expert surgeon. We confidently refer to the cases introduced, and Dr. O'Beirne's own description of the operation, for proof of the justness of these observations. Dr. O'B. ought not to be mortified if his favourite remedy be thus limited in the range of its application; for it will still be a very valuable addition to the list of our remedial agents, in difficult emergencies. We have little doubt that the author will be greatly offended with us for this "discouragement," as he will call it; but should the author and ourselves live for ten years longer, we hereby invite him to publicly impugn our prognostications, should they turn out false. In respect to his "new views of defecation," we fairly give him considerable merit for originality. We are not, indeed, entire converts to all his views; but we are free to confess that his work will tend to remove some erroneous views entertained by the great body of the profession respecting the function of the rectum and the sigmoid flexure of the colon. However, we have so fully delineated the contents of the book, that no misconception can result, even where the original, (which we strongly recommend) does not come under the cognizance of the reader.

